# Nucleotide Excision Repair: From Molecular Defects to Neurological Abnormalities

**DOI:** 10.3390/ijms22126220

**Published:** 2021-06-09

**Authors:** Yuliya Krasikova, Nadejda Rechkunova, Olga Lavrik

**Affiliations:** 1 Institute of Chemical Biology and Fundamental Medicine, Siberian Branch of the Russian Academy of Sciences, 630090 Novosibirsk, Russia; Y.Krasikova@gmail.com (Y.K.); nadyarec@niboch.nsc.ru (N.R.); 2 Department of Natural Sciences, Novosibirsk State University, 630090 Novosibirsk, Russia

**Keywords:** nucleotide excision repair, xeroderma pigmentosum, neurodegeneration, base excision repair, oxidative stress, mitophagy

## Abstract

Nucleotide excision repair (NER) is the most versatile DNA repair pathway, which can remove diverse bulky DNA lesions destabilizing a DNA duplex. NER defects cause several autosomal recessive genetic disorders. Xeroderma pigmentosum (XP) is one of the NER-associated syndromes characterized by low efficiency of the removal of bulky DNA adducts generated by ultraviolet radiation. XP patients have extremely high ultraviolet-light sensitivity of sun-exposed tissues, often resulting in multiple skin and eye cancers. Some XP patients develop characteristic neurodegeneration that is believed to derive from their inability to repair neuronal DNA damaged by endogenous metabolites. A specific class of oxidatively induced DNA lesions, 8,5′-cyclopurine-2′-deoxynucleosides, is considered endogenous DNA lesions mainly responsible for neurological problems in XP. Growing evidence suggests that XP is accompanied by defective mitophagy, as in primary mitochondrial disorders. Moreover, NER pathway is absent in mitochondria, implying that the mitochondrial dysfunction is secondary to nuclear NER defects. In this review, we discuss the current understanding of the NER molecular mechanism and focuses on the NER linkage with the neurological degeneration in patients with XP. We also present recent research advances regarding NER involvement in oxidative DNA lesion repair. Finally, we highlight how mitochondrial dysfunction may be associated with XP.

## 1. Introduction

In all cells, DNA is the carrier of genetic information from generation to generation; thus, its integrity must be maintained to ensure the survival of the cell, the whole organism, or even the whole species. Nonetheless, DNA is constantly jeopardized by multiple external adverse factors, such as ultraviolet (UV) light, ionizing radiation, chemotherapy drugs, or environmental pollutants. DNA damage can also be caused by endogenous factors such as replication errors or cellular oxidative metabolism products from mitochondria or inflammation [1]. The lesions can disrupt the basic processes of DNA metabolism by blocking replication and transcription. To counteract these adverse effects, eukaryotic cells are equipped with several DNA repair mechanisms acting on different types of DNA damage [2,3]. In some cases, if the lesions cannot be eliminated—either because the damage load is too high or because a requisite repair pathway is deficient—the cell cycle can be arrested until the damage is repaired, and if this does not occur rapidly, the cell may be eliminated by apoptosis or may accumulate mutations and transform into a potentially cancerous cell that might proliferate uncontrollably and give rise to a tumor. Ultimately, cells can tolerate some DNA lesions owing to translesion DNA synthesis. 

Typical types of DNA lesions include a variety of oxidative DNA modifications involving base or sugar damage, DNA crosslinks, strand breaks, and adducts with chemically active molecules [2,3]. Moreover, DNA can be damaged because of internal instability due to the spontaneous hydrolysis of the glycosidic bond with the formation of an abasic site (i.e., apurinic/apyrimidinic site, hereafter: AP site). As a rule, base excision repair (BER) deals with the repair of nonbulky base damage and AP sites in both the nuclear and mitochondrial cellular compartments [1,4,5]. 

The nucleotide excision repair (NER) pathway is the most universal repair pathway to remove a wide range of helix distorting lesions from DNA [6,7]. NER substrates are UV photoproducts, e.g., cyclobutane pyrimidine dimers (CPDs), pyrimidine-pyrimidone-(6-4)-photoproducts (6-4PPs), intrastrand crosslinks, and bulky adducts of DNA bases with reactive metabolites of some chemical carcinogens or chemotherapeutic agents [8]. These kinds of lesions can be substrates for two NER sub-pathways—global genome NER (GG-NER) and transcription-coupled NER (TC-NER)—that overlap, except for the mode of DNA damage recognition. Specific damage sensing proteins of GG-NER scan the entire genome at any moment of the cell cycle [6,7,8]. In contrast to GG-NER, TC-NER rapidly eliminates transcription-blocking lesions from actively transcribed DNA strands only. During TC-NER DNA damage can be detected in the template DNA strand when it stalls the RNA polymerase [9,10]. After the lesion has been recognized, all subsequent steps require the same NER core factors in GG-NER and TC-NER.

Mutations in NER-related genes cause several hereditary diseases, such as xeroderma pigmentosum (XP) and Cockayne syndrome (CS) [11]. Mutations in XP-related gene products (except proteins that exclusively taking part in GG-NER damage recognition) lead to the disruption of both NER sub-pathways. At the same time, mutations in CS proteins affect only TC-NER. XP is characterized by extreme sensitivity of the skin to sunlight and a dramatically increased risk of skin cancer [12,13]. A subset of XP patients develops a profound neurodegenerative condition known as XP neurological disease [14]. XP and CS are often grouped together as related diseases owing to overlapping sun sensitivity phenotypes and progressive neurodegeneration, but the specific nature of the neurological pathologies is qualitatively different between them [15,16,17]. 

Progressive neurodegeneration occurs when a loss of neuronal structure or function leads to a decline in the number of neurons owing to apoptotic cell death [2]. Neurons have a high metabolic load and consume large amounts of energy, which is supplied by mitochondria in the form of ATP. Byproducts of the ATP formation give rise to reactive oxygen species (ROS), which can cause many types of oxidative DNA damage to genomic and mitochondrial DNA [18,19]. Nowadays, it is widely accepted that the accumulation of oxidative DNA lesions is the cause of the neuropathology that takes place with aging as in several neurodegenerative disorders. Moreover, the accumulation of damaged mitochondria due to a decrease in mitophagy is also a hallmark of the aging process and a clinical feature of XP and CS [18,20,21].

This review focuses on the link of NER with the neurological disease in patients with XP. Firstly, we provide general information about the molecular mechanism of NER. Next, we briefly review the clinical features of XP disorder and discuss the clinical and neuropathological characteristics of XP neurological disease. Then, we overview the involvement of NER in the repair of oxidative DNA lesions and discuss the specific class of oxidatively induced DNA lesions, 8,5′-cyclopurine-2′-deoxynucleosides, which are considered as the endogenous DNA lesions responsible for the neurological problems in XP patients. Finally, we describe the way in which mitochondrial dysfunction is believed to be associated with XP.

## 2. Nucleotide Excision Repair 

### 2.1. Classic NER Substrates

One of the most astonishing features of the NER pathway is its broad ability to recognize and process many structurally and chemically diverse lesions. NER is the only repair pathway that protects our skin from DNA photodamage induced by UV light. The latter is the high-energy component of sunlight that reaches the Earth surface. According to the wavelength, UV radiation can be subdivided into several ranges. Fortunately, the atmosphere blocks ~3/4 of the sun’s UV light, and its most powerful part, UV-C (100–280 nm), is completely absorbed. The ozone layer filters most of UV-B (280–315 nm). Thus, most of the UV light that reaches the Earth surface is UV-A (315–400 nm) with a small remainder of UV-B. UV-A can penetrate more deeply into the skin than UVB can because of its longer wavelength. Photodamage formation (and sunburn) in human skin starts near the boundary between UV-A and UV-B light (~315 nm) and continues during UV-B exposure. Notably, a wavelength closer to the nucleotide light absorption maximum produces more lesions in DNA.

**Figure 1:**
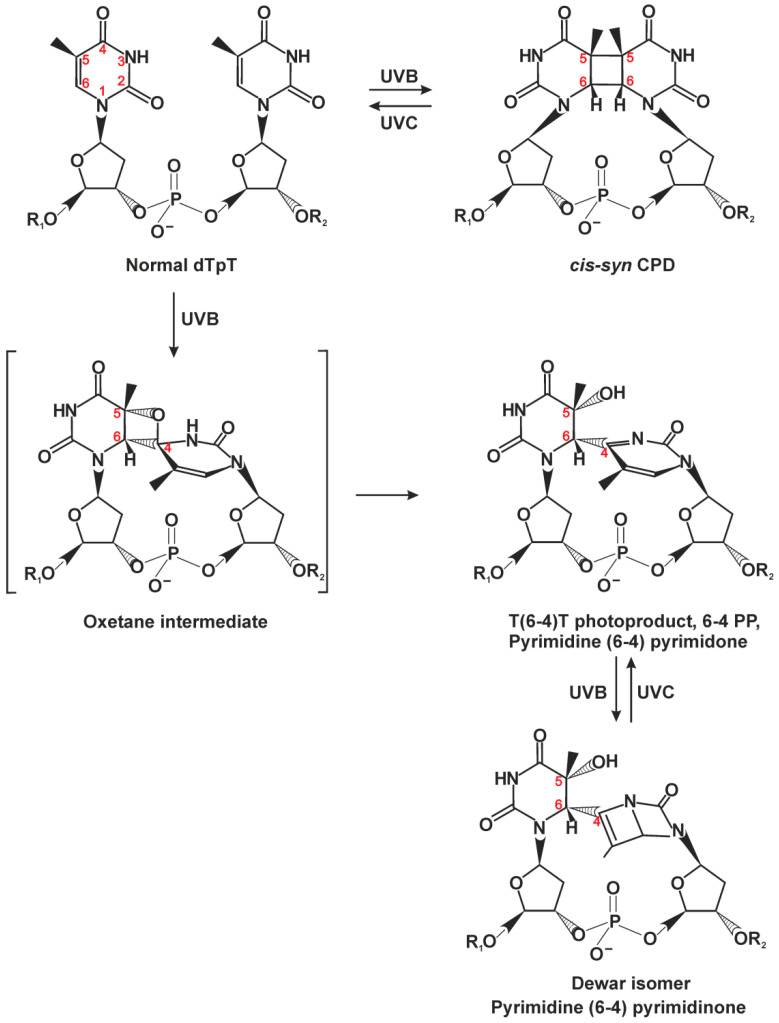
Chemical structures of DNA photoproducts caused by sunlight. The majority of cyclobutane pyrimidine dimers (CPDs) is formed between adjacent thymine residues (TT) but can eventually arise between adjacent T and C, C and T, or C and C, depending on the wavelength, irradiation dose, and adjacent sequences. CPD can be formed with *cis-syn* isomer representing large majority of CPDs within duplex DNA, and *trans-syn* occurring exclusively within single-stranded DNA. Pyrimidine-(6-4)-pyrimidone photoproducts (6-4PPs) are generated preferentially in nucleotide pairs TC, CC, and TT, with the ratio and yields depending on irradiation wavelength and adjacent sequences [8]. This figure is based on several studies [8,22].

CPDs are the major DNA photoproducts of UV light (Figure 1) [7,8,22,23]; 6-4PPs are formed in a 25–30% lower amount than CPDs and are the second most prevalent UV lesion. CPDs only minimally distort the double helix, whereas 6-4PPs produce a pronounced DNA backbone bending and base-pairing disruption. The DNA thermodynamic destabilization ability correlates with repair efficiency of these lesions; CPDs are excised by NER with much slower kinetics as compared to 6-4PPs [1,22,24,25].

Another source of DNA damage is various electrophilic compounds that directly penetrate the cell from an external medium or are produced inside the organism after metabolic activation. These compounds include environmental mutagens like polycyclic aromatic hydrocarbons (benzo[*a*]pyrene and various aromatic amines) and adducts of cancer chemotherapeutic drugs such as cisplatin. These electrophilic compounds can react with nucleophilic atoms of DNA, especially with N7 of the guanine base [7,8]. 

DNA damage recognition is the first key step, which affects overall efficiency of DNA repair [6]. The fact that NER can repair so many structurally different types of DNA damage indicated early on that the system may not recognize a lesion per se but rather some specific conformational features caused by the lesion within DNA [7]. In general, a good NER substrate should be bulky and must destabilize a DNA double helix (disrupt base pairing and bend the duplex). To detect both conditions, NER has evolved special bipartite substrate discrimination: firstly, it recognizes a local thermodynamically destabilized site, and then the latter is probed for lesion presence. The double recognition allows NER to avoid processing mismatched but damage-free sites.

### 2.2. The Damage Recognition Step

DNA damage can be recognized by NER in one of two modes. GG-NER can search for damage anywhere in the genome throughout the cell cycle. The second mode is TC-NER, which is responsible for the accelerated repair of lesions in the template DNA strand of actively transcribed genes only.

In the case of mammalian GG-NER, lesions are recognized by xeroderma pigmentosum factor C (XPC) complexed with proteins RAD23B and centrin 2 (CETN2) [6]. Small subunits collectively stabilize the XPC structure, possibly modulate some protein–protein interactions, and stimulate the DNA binding of the major subunit of the complex (XPC) thereby increasing NER efficiency in vitro and in vivo ([26] and references within). The XPC–RAD23B–CETN2 complex (hereinafter, XPC) can detect and bind DNA sites where the regular double-helical structure is perturbed, and as a result, one or more base pairs are disrupted and/or destabilized [6,7]. X-ray crystal structure of Rad4—the yeast ortholog of XPC—has revealed a structural basis for the unique DNA damage-searching ability [27,28]. A series of subsequent biophysical studies indicates that Rad4/XPC can bind to DNA nonspecifically via a damage-independent DNA-binding domain (TGD) and freely diffuse mainly by a one-dimensional-diffusion mechanism [7,29,30]. It is noteworthy that Rad4/XPC diffuse along DNA not by “sliding” but rather by “hopping” (diffusion through repeated microscopic dissociation and reassociation with the DNA). An advantage of the hopping mode is that it allows a protein to overcome protein obstacles on DNA [29]. At a suspicious DNA site of certain single-stranded character (where DNA is “breathing” too much because of a mismatch or an AT-rich sequence, where DNA can transiently melt), Rad4/XPC are slowed down [29,30]. The presence of a helical distortion and base pair disruption enables XPC to insert two β-hairpin modules from BHD2/BHD3 domains into the DNA duplex and to form a stable protein–DNA complex. In this complex, Rad4/XPC interacts exclusively with the nucleotides on the undamaged strand and flips out damage-containing nucleotide pairs to form an “open” conformation [30]. The reason is that the damaged DNA is already destabilized and has a lower free-energy barrier for “opening,” thus increasing the probability that Rad4 (or XPC) can use the hairpin modules to sense the lesion presence and not diffuse away [7,30]. Notably, DNA duplexes containing bulky lesions on both strands are not processed by NER [31,32].

**Figure 2:**
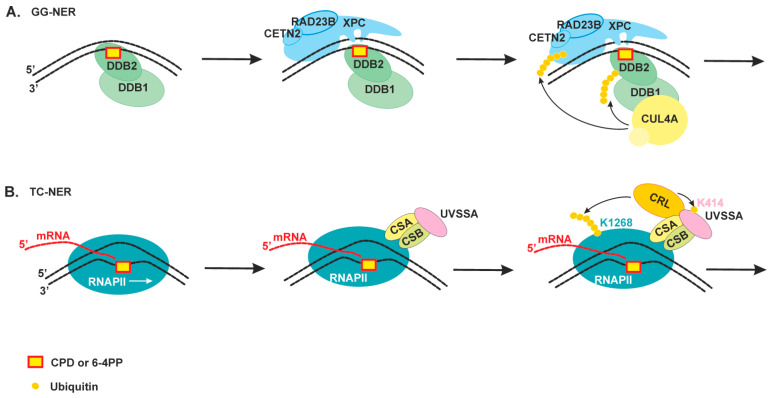
An overview of the damage recognition step of nucleotide excision repair (NER). (**A**) Global genome NER (GG-NER) can search for damage anywhere in the genome throughout the cell cycle. The UV-DDB protein recognizes CPD or 6-4PP, directly binds to it through its DDB2 subunit, and facilitates efficient recognition of the lesion by the XPC–RAD23B–CETN2 complex. The DDB1 subunit is also a connector protein for ubiquitin ligase CUL4, which ubiquitinates DDB2 and XPC [33]. (**B**) Transcription-coupled NER (TC-NER) is responsible for accelerated repair of lesions in the template DNA strand of actively transcribed genes only. The CSB protein and then proteins CSA and UVSSA bind to DNA damage stalled RNAPII. CSB and CSA associate with CRL ubiquitin ligase and contribute to the ubiquitination of the RNAPII RPB1 subunit at K1268. This ubiquitination stimulates the association of TFIIH with the stalled RNAPII through a transfer mechanism that also involves UVSSA-K414 ubiquitination [9,35].

CPDs—the most abundant photolesions—are poorly recognized by XPC because they cause only a minimal distortion in DNA. These lesions are recognized by a special protein (UV-damaged DNA-binding protein (UV-DDB), a heterodimeric protein consisting of DDB1 and DDB2/XPE), which has extraordinarily high binding affinity and specificity for CPD and 6-4PP [6,7]. In contrast to XPC, DDB2 interacts directly with UV light–induced photolesions in DNA, introduces a kink into the duplex, and creates a more suitable substrate for XPC (Figure 2A). Structural studies have revealed that DDB2 flips out the two nucleotides of CPD into a shallow binding pocket, which can accommodate such lesions as CPDs or 6-4PPs via shape complementarity [6,7]. In addition, DDB2 is thought to facilitate XPC recruitment within chromatinized DNA through the ability to promote chromatin reorganization. Moreover, DDB1 is also a connector protein for ubiquitin ligase CUL4–RBX1 [7,33]. The ubiquitin ligase is activated upon DDB2 binding and ubiquitinates DDB2 and XPC [33]. The ubiquitination of DDB2 launches its proteasomal degradation after extraction from NER complexes. By contrast, the XPC ubiquitination increases its DNA-binding activity [34]. Damage handover from DDB2 to XPC coincides with the arrival of the TFIIH complex, which further promotes DDB2 dissociation [33,35]. It should be noted that both UV-DDB and XPC proteins are also targets of poly(ADP-ribosyl)ation catalyzed by poly(ADP-ribose)polymerase 1 (PARP1) in response to UV-irradiation. Taking in account that PARP1 participates in the UV-induced chromatin decondensation and PARP1 activity promotes DDB2 interaction with XPC, this modification can facilitate lesion recognition in the chromatin context (reviewed in [36]).

TC-NER is initiated by the stalling of elongating RNA polymerase II (RNAPII) at DNA lesions (Figure 2B). The CSB protein (Cockayne syndrome group B protein, a member of the SNF2 family of DNA-dependent ATPases) interacts loosely with the elongating RNAPII and stimulates transcription but becomes more tightly bound after transcription arrest [10]. It is suggested that CSB participates in RNAPII backtracking to make a DNA lesion accessible to repair proteins. Upon RNAPII stalling at a lesion, the RNAPII-bound CSB recruits the CSA protein (Cockayne syndrome group A protein), and both together contribute to the polyubiquitination of the K1268 residue of RPB1, a subunit of RNAPII [35,37,38]. The RPB1 ubiquitination acts as a master switch for the alternation of transcription, RNAPII degradation, and initiation of DNA repair [9]. At the next step, CSA facilitates the association of UVSSA (UV-sensitive syndrome protein A) with the stalled RNAPII. UVSSA is the key factor that recruits the TFIIH complex [37].

When the RPB1 K1268 residue is mutated or some of CSB/CSA/UVSSA accessory proteins are absent, TC-NER cannot start. In this situation, transcription does not shut down, leading to multiple transcription restarts (resulting primarily in the transcription of short genes) and subsequent RNAPII stalling instances; therefore, eventually, the RNAPII pool is depleted, and transcription is dysregulated ([38] and reviewed in [9]). 

A recent study has discovered that RNAPII stalling could follow by nascent RNA hybridization with DNA template strand generating an RNA–DNA hybrid and displaced ssDNA. Such kind of structures called an R-loop could occur physiologically during an early step in transcription elongation (especially are abundant at promoters) and transcription termination [39,40]. Moreover, it is proposed that R-loops can regulate gene expression through multiple context-dependent mechanisms. At the same time, R-loop can be problematic for cells as it blocks efficient transcription and replication. The accumulation of R-loops is associated with cancer and several neurological diseases. It was shown that NER proteins participate in R-loops resolving process, but the mechanism of R-loop resolution is not clear. The TFIIH (transcription factor IIH) complex is a multifunctional protein machine required for transcription initiation and NER [41]. Depending on a context, its composition changes from a core of seven subunits, including the XPB translocase and XPD helicase, to 10 subunits, through the addition of three CAK (Cdk-activating-kinase module) kinase subunits. Recent advances in breakthrough cryo-electron microscopy give investigators a unique opportunity to investigate the TFIIH structure [41,42,43]. TFIIH assumes an arch-like conformation with subunits curving from XPD on the one end to XPB located on the second end. The CAK components close the ends of this structure and stabilize the arch. It is suggested that TFIIH core structure becomes more flexible after CAK module dissociation, and this arrangement may be sufficient for subsequent functioning during NER [7]. The release of the CAK complex from core TFIIH transforms TFIIH from a transcription factor into a repair factor [37].

### 2.3. Damage Verification and Pre-Incision Complex Formation

The TFIIH complex is the key protein for the damage verification step. TFIIH probes the lesion itself and unwinds the DNA duplex around the lesion, thereby making room for the subsequent assembly of a repair machine; we simply could even say that NER machinery is built around TFIIH.

**Figure 3:**
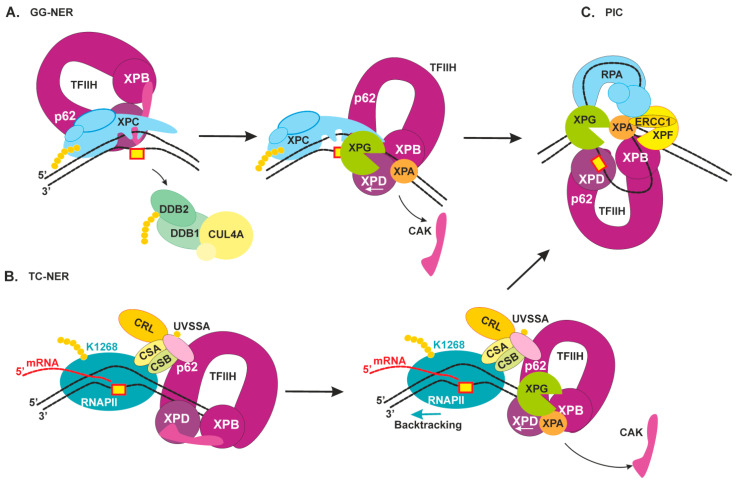
Schematic view of the damage verification step of NER and pre-incision complex formation. (**A**) GG-NER. TFIIH initially interacts with XPC’s N terminus by means of p62 subunits, then tumbles to XPC’s C terminus, where the interaction with XPB promotes its binding to a duplex part. XPA releases an inhibitory CAK module and together with XPG stimulates XPD activity [45]. The XPD helicase binds to the damaged strand and starts to a repair bubble formation [7]. When XPD gets to the lesion and stalls on it, XPC is displaced, and XPG binds to the 3′ edge of the repair bubble. (**B**) TC-NER. XPC and UVSSA share an interaction surface on the p62 subunit of TFIIH [41]. RNAPII moves in the 3′→5′ direction on the damaged strand, then, after its lesion stalling and assembly of factors CSB, CSA, and UVSSA, the latter promotes TFIIH binding downstream of RNAPII [37]. Thereafter, XPA and XPG stimulate XPD activity, and TFIIH starts to move in the 5′→3′ direction and may “push” RNAPII for a backtracking movement. (**C**) The NER pre-incision complex (PIC): TFIIH stalls on the lesion-bearing strand, RPA covers the undamaged strand, XPA marks the 5′ edge of the repair bubble, XPG marks the 3′ edge of the repair bubble, and XPF–ERCC1 binds behind XPA.

In the case of GG-NER (Figure 3A), TFIIH is recruited via XPB engagement to the DNA duplex and interaction with the C terminus of XPC as well as an additional interaction of the p62 subunit with XPC’s N terminus [7]. After that, the XPD helicase may get loaded on the DNA because of its location on the other end of the TFIIH arch. A striking similarity between GG-NER and TC-NER is that XPC and UVSSA share an interaction surface on the p62 subunit of TFIIH, suggesting that the two pathways at least partially share a mechanism for the engagement of TFIIH with the lesion site [41]. 

Notably, human TFIIH binds downstream of RNAPII (which moves in the 3′→5′ direction) in the transcription pre-initiation complex [44]. In line with these data, we propose that in TC-NER initiation, TFIIH should also bind downstream of RNAPII for subsequent movement on the same damaged strand in the opposite 5′→3′ direction (Figure 3B). In the case of R-loop formation behind the RNAPII, it cannot be easily displaced by TFIIH to make a space for repair process. The sequence of events in this situation should be a subject for future investigations.

TFIIH structure flexibility allows XPD to unwind DNA while tracking along in the 5′→3′ direction [7]. During the tracking process, XPD pulls the DNA through a narrow tunnel that is too small for bulky DNA lesions to pass through [7]. This “damage filtration” process is simple but effective. 

The release of the CAK module from core TFIIH is triggered by the association of repair factors XPA and XPG (XP factors A and G) [45]. Biochemical data show that XPA can stimulate the overall helicase activity of TFIIH, and on the contrary, can inhibit the helicase activity in the presence of lesions; therefore, XPA also contributes to damage verification [46]. Moreover, XPA has some bulky-damage recognition ability as well and especially prefers to bind kinked and branched DNA structures [47,48]. It was shown recently by atomic force microscopy, scanning force microscopy, and mathematical modeling that XPA undergoes episodic one-dimensional diffusion to search DNA for damage [49]. Furthermore, biochemical research revealed that XPA is located on the 5′ side from a lesion at the damaged bubbled DNA [47]. Cryo-electron microscopy data have extended our knowledge of the modulation of TFIIH activity by XPA and XPG [45]: (1) XPA and XPG stabilize an alternative conformation of TFIIH, where the XPD helicase is opened for functioning; (2) XPA and XPG also stimulate XPB and XPD, and this event may facilitate DNA opening; consequently, they are present in a ternary complex in the lesion-scanning mode; (3) XPA interacts with an XPB subunit in the TFIIH–DNA complex and marks the DNA at the 5′ edge of the repair bubble; (4) XPA forms a bridge between XPB and XPD and thus possibly facilitates XPD positioning on the single-stranded 3′ extension.

Immediately after forming single-stranded undamaged DNA inside the repair bubble, it binds to the replication protein A (RPA) [50]. RPA interacts with the undamaged strand and protects it from a nuclease attack [47]. The size of the NER-excised fragment coincides with maximal length of the single-stranded–DNA platform for RPA binding (approximately 30 nt), to which RPA binds tightly with defined 5′→3′ polarity [51]. RPA tightly interacts with XPA inside the repair bubble, and they together regulate the correct orientation and activation of NER nucleases [52]. Moreover, an ability of RPA and XPA to form a complex in the absence of DNA as well as a ternary complex with DNA was reported, and XPA interaction with RPA is indispensable for NER [51]. Crystal structure of *Ustilago maydis* RPA stably bound to single-stranded DNA was resolved some time ago [53]. These data revealed that single-stranded DNA in complex with RPA is also U-shaped; for this reason, the 5′ edge and 3′ edge of the repair bubble are pulled together. 

During the lesion scanning by TFIIH, XPG “rides” on the XPD subunit [45]. After XPD stalls on the lesion, XPG binds to the 3′ edge of the repair bubble (possibly by simultaneous displacement of XPC). NER pre-incision complex formation is completed by the engagement of XPF–ERCC1, which is recruited by XPA [54]. 

Thus, the interior of the NER pre-incision complex is as follows: TFIIH stalls at the lesion, RPA covers the undamaged opposite strand, XPA marks the 5′ edge of the repair bubble, XPG marks the 3′ edge of the repair bubble, and XPF–ERCC1 binds behind XPA. The XPA is a central component in the pre-incision complex room because it interacts with all its compartments: with the damage recognition proteins XPC and DDB2, verifies the damage and interacts with TFIIH and RPA, promotes correct positioning of both nucleases. Patients with reported mutations in the *XPA* gene have the severest form of XP (we discuss it in the next chapter). Today, XPA is considered as organizing or scaffold component of the pre-incision complex, which makes sure that all the NER factors are in the right place for the incision to occur [23,48]. 

### 2.4. Dual Incision, Resynthesis, and Ligation

**Figure 4:**
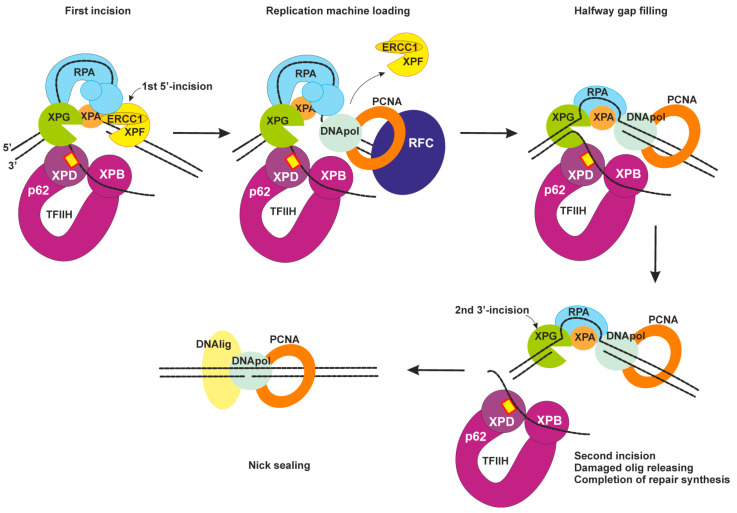
Late NER stages: dual incision, resynthesis, and ligation. The first incision is carried out by XPF–ERCC1 from the 5′ side to the lesion site. Then, replication machinery is loaded, and repair synthesis can be initiated. Possible sets of replication machines: DNA polymerase δ, PCNA, and RFC; DNA polymerase ε, PCNA, and a modified form of RFC; or DNA polymerase κ, ubiquitinated PCNA, and XRCC1. Halfway gap resynthesis is followed by a second incision by XPG. After repair synthesis is completed, nick sealing is performed by DNA ligase I or by the DNA ligase IIIα–XRCC1 complex.

Two endonucleases XPF–ERCC1 and XPG can now incise the lesion-containing DNA strand (Figure 4). The DNA incision is first carried out by XPF–ERCC1 from the 5′ side to the damage site with the formation of a free 3′-OH group [55]. Next, replication machinery can be loaded to start repair synthesis [56]. RPA promotes the arrival and positioning of RFC and enhances repair synthesis with possible help of XPA as it interacts with PCNA [57,58]. 

Repair synthesis can proceed halfway through the gap in the absence of an XPG-made incision [23]. The XPG-made 3′ incision is possibly triggered by PCNA–XPG interaction [59]. The lesion-containing oligonucleotide (~30 nt) is released from the repair bubble in complex with TFIIH [60]. Then, after ATP binding, TFIIH slowly dissociates from the excised oligonucleotide, and the latter is bound by RPA or degraded by cellular nucleases.

Repair synthesis may be performed by different sets of replication machines: DNA polymerases δ/sliding clamp PCNA/clamp loader RFC or DNA polymerases ε/PCNA/ a modified form of RFC or DNA polymerases κ/ubiquitinated PCNA/XRCC1 [23,61]. Which set of replication factors will be loaded possibly depends on the cell cycle but in general remains unknown. The XPG-made incision leaves a 5′-phosphate that is utilized in the nick-sealing reaction by DNA ligase I or by DNA ligase IIIα (with XRCC1) [62]. Now, NER is completed.

## 3. The Molecular Basis of Xeroderma Pigmentosum

Efficient DNA repair is incredibly important for the health of an organism. Mutations in NER genes can result in a genetic disorder called xeroderma pigmentosum (XP), which is characterized by extreme UV sensitivity, abnormal skin pigmentation, and a dramatically increased risk of skin cancer in sun-exposed mucocutaneous areas and ocular structures. XP is an autosomal recessive genetic disorder that is extremely rare in Europe and North America (frequency of ~2.3 per million live births), but its prevalence is higher in Japan (frequency of 1 per 22,000), the Middle East, North Africa, and India [13,63]. Corresponding to causative mutation proteins, all XP cases are divided into clinically heterogeneous complementation groups (CGs): XP-A to XP-F and variant form XP-V. Patients with mutations in one XP gene belong to one CG. Patients with CG XP-V bear mutations in the bypass polymerase *POLH* gene encoding DNA polymerase ƞ.

The skin of affected individuals is normal at birth, but then, usually within first weeks of life, the infants can get sunburn after minimal exposure to sunlight (~60% of cases). In the other cases (~40%), the infants have a normal acute response to sun exposure. Nevertheless, they all develop an unusually high number of freckle-like pigmentary changes in sun-exposed areas, often by 2 years of age (Table 1) [12,64]. The next clinical manifestations are malignant skin neoplasms on the face, neck, and upper trunk. The average age of the first skin cancer is less than 10 years, and a patient can develop hundreds of skin cancers [12]. It has been estimated that XP patients have a 10,000-fold higher risk of basal and squamous cell carcinomas and a 2000-fold higher risk of malignant melanoma before the age of 20 [63,65]. Besides, XP patients exhibit a greatly increased frequency of cancer of the oral cavity, particularly squamous cell carcinoma of the tip of the tongue [12].

CGs XP-A and XP-C are more common (approximately 50% of patients), and XP-F is one of the rarer CGs [13,63]. Six years ago, a study was published with detailed clinical and molecular information on the largest analyzed cohort of XP patients [13]. This paper revealed clinical symptoms differences among XP CGs and heterogeneity within each CG. Moreover, the clinical features are strongly dependent on distinct locations and types of mutations in the causative genes [13]. Most patients in CGs XP-A, -B, -D, -F, and -G present with severe sunburn reactions starting from an early age (Table 1). In contrast, TC-NER–competent groups XP-C, -E, and -V have normal sunburn reactions for a skin type. Paradoxically, those XP-C, -E, and -V patients get an XP diagnosis at a later age and therefore accumulate more photodamage, meaning an earlier age of the first skin cancer. XP-F and XP-G patients seem to be remarkably resistant to the development of skin cancers as compared to other XP CGs. 

At least 40% of patients have ocular diseases, but they are strikingly limited to the anterior, UV light–exposed structures of the eye. Blepharospasm and photophobia are common symptoms, and continued sunlight exposure may result in severe keratitis and cancers (epithelioma, squamous cell carcinoma, and melanoma) [63,64]. XP-C patients are especially susceptible to ocular problems [13]. In addition to the common XP symptoms, XP patients under the age of 20 have an approximately 50-fold higher prevalence of cancers of the brain and other parts of the central nervous system. The reason for brain cancer predisposition is currently poorly understood [12].

## 4. XP Neurological Disease

Between 20% and 30% of XP patients have neurological problems referred to as XP neurological disease (Table 1) [66]. Such infants are normal at birth, and the clinical manifestations may start between 2 years and a middle age [64]. The earliest signs of the disease are reduced tendon reflexes and high-frequency sensorineural hearing loss, and these can be employed in screening tests [12]. In some cases, the affected individuals demonstrate a delay in developmental milestones. The disease progresses to uncoordinated movements (ataxia) with subsequent loss of the ability to walk, and eventually, the patient becomes wheelchair bound. Other clinical features of these patients may include a loss of the ability to swallow, areflexia, microcephaly, and progressive cognitive impairment [64]. Some XP patients also show progressive neurodegeneration with some features of premature aging [14].

The progressive neurological abnormalities are seen primarily in XP patients belonging to the CGs in which both NER subpathways are compromised (Table 1): XP-D and XP-A (followed by XP-B, XP-G, and XP-F) [13,14,64,65]. Patients belonging to the CGs with intact TC-NER (XP-C, -E, and -V) in general do not have neurological problems [14]. Nonetheless, there are some data suggesting that XP-C associates with the development of intracranial lesions [13]. Accordingly, we can conclude that the TC-NER pathway is essential for neurons, in contrast to the GG-NER pathway whose deficiency leads only to mild neurodegeneration. By means of the NT2-hNT human cell system, it was found that terminally differentiated neurons can have another repair phenotype as opposed to mitotic cells [67]. The GG-NER activity in these post-mitotic neurons is greatly impaired, whereas the TC-NER activity is normal. This observation was partially confirmed on cultured neurons and astrocytes derived from rat embryonic brains that have significantly lower NER capabilities than fibroblasts do [68].

These neurological abnormalities are due to a progressive atrophy of the brain, spinal cord, and peripheral nervous system. The pathology is primary neurodegeneration without evidence of other obvious causative processes [14]. This progressive neurodegeneration is due to apoptotic neuronal death. Given that terminally differentiated neurons no longer divide, apoptosis may result in an uncompensated cell loss for the organism, and up to 40% of brain tissue mass can be lost [15]. This finding is supported at the histological level by the observation that losses of neurons occur in several different regions of the brain [2]. Large neurons appear to be more strongly affected by the degeneration than smaller neurons. The peripheral nervous system is also frequently involved in the degenerative process. 

XP patients with neurodegeneration have poorer survival rates than XP patients without. The median age of death of the affected patients has been reported to be 29 years, as compared to 37 years for the patients without neurodegeneration. The most common causes of death are skin cancer (34%), neurodegeneration (31%), and internal cancer (17%) [14].

XP overlaps with CS in terms of sun sensitivity phenotypes (Table 1). CS is also a rare autosomal recessive disease caused by mutations in the *CSB* gene (CS-B patients, 62% of cases) or in *CSA* (CS-A patients), whereas certain mutations in *XPB*, *XPD*, or *XPG* yield a disease with combined features of CS and XP [11,69]. CS clinical features often include photosensitivity and cataracts as well as an abnormal “bird-like” facies and severe cachectic dwarfism; special hallmarks are profound postnatal growth failure of the soma and brain associated with premature senescence and progressive multiorgan degeneration [2,11]. The neurological symptoms include demyelination in the cerebral and cerebellar cortex, calcification in basal ganglia and cerebral cortex, neuronal loss, sensorineural hearing loss, and decreased nerve conduction. CS patients’ brains show an unusual severe patchy myelin loss (“tigroid leukodystrophy”) and segmental demyelinating peripheral neuropathy. This specific type of pathology is not observed in XP or in any other neurological diseases associated with defective DNA repair. In addition, a calcification of basal ganglia and other regions of the brain is observed in CS but not in XP [2,11,15,70]. The disease does not seem to confer an increased risk of cancer. The life expectancy of CS patients is 12.5 years, and because many of the disease’s signs resemble normal aging, it has been classified as premature aging syndrome.

## 5. Neurological Abnormalities Due to High Oxidative DNA Damage in Neurons

The reasons for neurodegeneration are not well understood. Clearly, there cannot be a direct connection between neurodegeneration and UV light exposure: sun UV radiation cannot penetrate the skull. At the same time, we know that the blood–brain barrier (BBB) protects the central nervous system from many exogenous substances, even though part of chemical compounds, probably at lower concentrations, can normally pass the BBB [71] Chemotherapy agents (such as cisplatin) could pass through the BBB and cause chemotherapy-related cognitive impairment because of hippocampal synaptic damage and neural cell loss [72,73]. Moreover, the BBB structure and functioning is also reduced with age [71,74]. Age-related BBB permeability increasing could lead to additional central nervous system damage and eventually to neurodegeneration. Anyway, as we mentioned above XP neurological disease could manifested in very young age, so we do not have enough data about this disease linkage with the BBB impairment and chemotherapy toxicity.

More than 40 years ago, it was proposed that some types of endogenous DNA damage may arise in the brain that are repaired by NER, and that a deficiency in this repair pathway causes damage accumulation and subsequent neurodegeneration (reviewed in [75]). A similar neurodegenerative process due to permanent neuron loss may proceed with aging. The “free radical theory of aging” postulates that accumulation of unrepaired oxidative damage leads to a cellular decline and associated age-related deterioration [2,21]. This “aging” theory for XP neurodegeneration is supported by the findings of Lindahl and colleges indicating that DNA exposure to ROS generates a class of DNA lesions that are normally repaired by NER (reviewed in [15]). They have proposed that oxidative stress may be the source of the DNA damage that causes neuronal death in XP patients. Thus, the search for this damage type has become an area of active research.

### 5.1. NER Impact in Oxidative Lesions Repair

It is known that neurons have a high metabolic load, consume large amounts of molecular oxygen, and need large amounts of energy from mitochondria. The ROS that are normally produced during cellular respiration can cause many types of oxidative DNA damage. Moreover, neurons are terminally differentiated post-mitotic cells; consequently, unrepaired oxidative DNA lesions accumulate over time and eventually can cause neuronal death. It is generally accepted that a wide spectrum of nonbulky oxidative DNA lesions is repaired by the BER pathway that is not deficient in XP. To summarize, current theories suggest that NER involved in the repair some types of oxidative DNA damage, and a lack of its repair function results in DNA damage accumulation, which is thought to be the reason for neurodegeneration in XP CGs.

**Figure 5:**
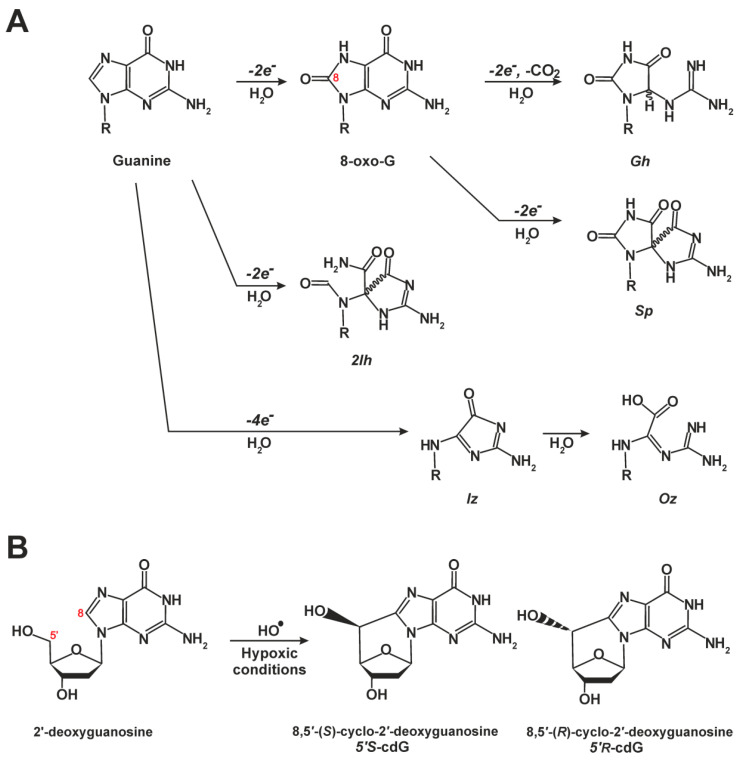
Chemical structures of common oxidative DNA lesions. (**A**) Guanine oxidation products. Products of two-electron guanine oxidation are 8-oxo-7,8-dehydroguanine (8-oxoG) and 5-carboxamido-5-formamido-2-iminohydantoin (2Ih). Four-electron guanine oxidation products are 5-guanidinohydantoin (Gh), spiroiminodihydantoin (Sp), 2,5-diamino-4H-imidazol-4-one (Iz), and 2,2,4-triamino-5(2H)-oxazolone (Oz). (**B**) Formation of 8,5′-cyclo-2′-deoxyguanosine (cyclic guanosine, cdG) through hydroxyl radical (HO•) oxidation under hypoxic conditions.

The most easily oxidized target in DNA is the guanine base (Figure 5A) [76,77]. The major two-electron guanine oxidation product of this reaction is 8-oxoguanine (8-oxoG, also known as 8-oxo-7,8-dihydro-2′-deoxyguanosine), which is easily oxidized than the guanine moiety, thus yielding four-electron guanine oxidation products spiroimino-dihydantoin (Sp), 5-guanidinohydantoin (Gh). As we mention above, these kinds of nonbulky oxidative lesions are almost exclusively repaired by the BER pathway [3,4,77].

During BER, DNA intermediates are bound by one or several BER enzymes (BER mechanism is reviewed extensively, see [78,79,80,81] for example). Thus, a question arises: how NER can be involved in oxidative damage repair? Do NER proteins stimulate the activity of BER enzymes or compete for DNA? Another possibility is that NER works as a backup system for BER; these are situations when a lesion cannot be repaired by BER or there are too many lesions to be repaired by BER alone. There is a completely different hypothesis too: some type of a bulky oxidative DNA lesion exists that is repaired only by NER, and BER proteins do not participate in this repair [15].

The first answers were obtained in a study [66] showing that a minimal set of NER proteins can remove oxidative DNA lesions from DNA in vitro. Subsequent long-term studies on BER and NER processes demonstrated that these repair systems do not work as isolated pathways and that the structure of an oxidative lesion influences the role of NER proteins in the repair response to the oxidative damage. In some cases, both pathways work cooperatively, and NER proteins stimulate the activity of key BER enzymes [1,3,5,82,83,84,85,86,87,88,89,90,91], but in other cases, the two pathways compete during recognition of the same lesion [92,93,94,95]. The data about XPC function in glycosylase—BER oxidative base lesion sensors—stimulation [96,97,98] raise questions about the relevance of these interactions as far as they absence in XP-C CGs does not lead to neurological disease. It is possible to speculate that XP-C individuals carry out normal TC-NER, so they can develop neurological symptoms much later in comparison with other XP patients [66]. Anyway, nonbulky oxidative DNA lesions cannot block transcription by RNA pol II [99]; thus, the only way these lesions could cause neurodegeneration is through a progressive decrease in the amount of the encoded proteins.

The text following an equation need not be a new paragraph. Please punctuate equations as regular text.

### 5.2. Cyclopurines Are Bulky Lesions and Exclusive NER Substrates

The following body of interesting data has been obtained regarding a specific class of endogenous oxidative DNA lesions: 8,5′-cyclopurine-2′-deoxynucleosides (cyclopurines) (Figure 5B). The cyclopurines contain a covalent bond between C8 of the base and C5 of 2′-deoxyribose [100,101]. Both lesions—8,5′-cyclo-2′-deoxyadenosine (cdA) and 8,5′-cyclo-2′-deoxyguanosine (cdG)—exist as 5′*R*- and 5′*S*-diastereomers [102,103] and are chemically very stable [104,105]. Lesions cdA and cdG are formed endogenously by an attack of hydroxyl radicals on sugar moieties [106] and can be generated in DNA by ɣ-irradiation under anoxic conditions [107,108] or by transition metal ion–mediated Fenton reactions [109]. Recently, two studies shed the light on cyclopurine formation in cellular models at different oxygen tension levels [110,111]. XPA-deficient (EUE-siXPA) human embryonic epithelial cell lines were found to accumulate higher levels of cyclopurines under hypoxic (1% O_2_) compared to physioxic (5% O_2_) conditions, and Fe levels were significantly higher in these cells too [110]. Diastereoisomeric ratios 5′*R*/5′*S* turned out to be independent of oxygen concentration, being 0.32 for cdG and 2.94 for cdA in EUE-siXPA cells (nearly equal to those in wild-type [EUE-pBD650] cells). The total amount of cyclopurines measured in that study was 1.82–2.52 lesions/10^6^ nucleotides. Higher concentrations of cyclopurines are observed under hypoxic (1% O_2_) conditions in CSA- and CSB-deficient fibroblasts as compared to their normal counterparts [110]. Because the mammalian cell nucleus is a relatively poorly oxygenated compartment, a crucial role of H_2_O_2_ in cyclopurine formation has been suggested [13,112].

Currently, cyclopurines are considered the best candidates for the DNA lesions responsible for neurological disease in XP patients. According to this prediction, XP patients should show elevated levels of cyclopurine-type lesions in their brain neuron DNA. Recently, this prediction was confirmed in an *Xpa^−/−^* mouse model, where cdA accumulation with age was shown specifically in brain tissues [75]. A similar accumulation of cdA has been documented in cell culture research: XP-C keratinocytes are inefficient at removing (5′*S*)-cdA, (5′*R*)-cdG, and (5′*S*)-cdG over time [96] and CS-A human primary fibroblasts show defective repair of (5′*S*)-cdA [113].

Both cdA diastereomers can block DNA replication process by polymerase δ [100] and transcription by RNA polymerase II [101]. The blockage of RNA polymerase II and a high number of stalled replication forks may serve as a trigger for apoptotic neuronal death [35]. Moreover, cdA lesion in a transcription factor binding site can block the binding of a transcription factor and inhibit gene expression in XP cells [114].

The next criterion for a candidate neurodegenerative DNA lesion in XP is that the lesion should be a NER substrate. The authors [100] used HeLa cell extracts and circular DNA substrates bearing 5′S- or 5′R-cdA to demonstrate that the cyclopurine residues are repaired by the NER pathway. Cyclopurine repair efficiency was found to be 40–150-fold lower than that of a 1,3-intrastrand d(GpTpG)-cisplatin crosslink, which is a well-repaired NER substrate. Additionally, 5′*R*-cdA was corrected more efficiently (~3–4-fold) than the *S* isomer. A study was published concurrently [101] that revealed a strong correlation between defective NER and neurodegeneration in XP patients. The authors performed complementation experiments using extracts from different NER-deficient cell lines and demonstrated that the repair was mediated by NER. It is especially noteworthy that an XP-A (XP12BE) cell line was used, which was derived from an XP-A patient (XP2OS) who had severe neurological symptoms: progressive neurodegeneration beginning as a loss of deep tendon reflexes and progressing to hearing loss, inability to walk, and eventually difficulty swallowing [63].

Two research groups [100,101] tested the possibility that cyclopurines can be repaired by BER. Both groups were unable to detect any evidence of such a repair pathway in extracts from the rat brain and in human cell extracts. Experiments with purified human DNA glycosylases revealed that NEIL1, NEIL2, and OGG1 fail to hydrolyze a duplex containing S-cdA or S-cdG [115]. The presence of a covalent 8,5′ bond is the most likely reason for the failure of BER to remove the lesion because even if a glycosylase can cleave the glycosidic bond, the base will remain attached to the sugar phosphate backbone via the 8,5′ bond [15]. The NER pathway repairs S-cdG slightly better than S-cdA in HeLa cell extracts, and the repair efficiency depends on the base opposite to the lesion. The complex investigation with all four cyclopurines confirmed that R-isomers are repaired better than S-isomers [116]. This isoform preference is due to greater distortions caused in DNA backbone by the R-isomers compared to the S-isomers.

Taken together, the aforementioned cyclopurine data suggest that cyclopurine lesions are responsible for the neurological disease in XP patients: (1) cyclopurines are formed during endogenous processes; (2) cyclopurines accumulate with age especially in the brain; (3) these lesions are exclusive NER substrates, and NER absence leads to damage accumulation; and (4) cyclopurines can block transcription and replication.

## 6. A Mitochondrial Echo of the Nuclear DNA Repair Deficiency

Mitochondrial health is another piece of the XP neurodegeneration puzzle. Given that mitochondria are the cell’s energy stations, they are indispensable for cellular function. Energy production is driven by the activity of the electron transport chain (ETC) within the mitochondria, which generates a proton gradient across mitochondrial membranes. This proton gradient called mitochondrial membrane potential and its maintenance are necessary for the functioning of ETC complexes and for normal oxidative phosphorylation. Hyperpolarization or depolarization of the mitochondrial membrane is pathological because it underlies ETC dysfunction [65]. Indeed, the sensing of mitochondrial membrane potential is indispensable for mitochondrial quality control and the subsequent elimination of damaged mitochondria.

Mitochondrial dysfunction is believed to be associated with neurodegenerative disorders. Mitochondrial autophagy (mitophagy) is a cellular pathway facilitating the degradation of damaged or unnecessary mitochondria. Mitophagy regulatory genes are mutated in patients with juvenile or early-onset Parkinsonism. The loss of a protein that is master regulator of transcription of mitochondrial biogenesis genes is a well-known feature of Huntington’s disease [117]. Clinically speaking, the mitochondrial disease phenotype is most commonly observed in brain or muscle tissue. Neurons and muscles—because of their high energy demands—may be particularly vulnerable to changes in ATP production; thus, mitochondrial alterations may entail neurodegeneration or myopathies even though the mitochondrial defect may be present in all tissues [118]. Mitochondria constantly divide, fuse, and travel throughout the neuron, from the cell body to nerve terminals and synapses, where energy is especially needed. Aberrations in these mitochondrial dynamics are associated with Alzheimer’s disease progression [19,119,120].

The clinical features of XP patients (XP-A CG), just as those of CS-B, share substantial similarities with what is often observed in mitochondrial diseases. CSB-deficient cells show mitophagy defects yielding increased mitochondrial content, increasing the membrane potential and free-radical amounts, and raising oxygen consumption. On the other hand, there is no evidence of a NER pathway in mitochondria, but the proteins CSA and CSB are present inside mitochondria, where CSA is involved in mitochondrial BER and CSB may takes part in a transcription process [17]. It was reported recently that in cells depleted of CSA or CSB, the resultant mitochondrial dysfunction can be corrected by supplementation with NAD^+^ precursors [16]. Therefore, it is proposed that: (1) the origin of mitochondrial problems in CS is in the nucleus, where a DNA repair deficiency leads to damage accumulation and an intensive poly(ADP-ribosyl)ation (PARylation) process with subsequent NAD^+^ depletion that is observed in CS-affected cells [17] and (2) changes in the NAD^+^ level are one of the communication ways between a mitochondria and nucleus [18].

As mentioned above, XP-A patients have a well-pronounced pathological mitochondrial phenotype. At the same time, there is no evidence of XPA presence in mitochondria; therefore, we cannot explain the mitochondrial deficiency by a direct link with an XPA deficiency. Nevertheless, it has been demonstrated that XPA-deficient cells harbor impaired mitophagy, yielding increased mitochondrial content, increased mitochondrial superoxide production, and hyperpolarization of the mitochondrial membrane [20,118]. High and prolonged ROS production has been reported for cells not only from XP-A but also from XP-D and XP-C patients [65].

Because mitophagy by the PINK1–Parkin (respectively PTEN-induced putative kinase 1 and E3 ubiquitin ligase) pathway is regulated by mitochondrial membrane potential, the mitophagy impairment may be caused by a deficiency of uncoupling proteins (UCPs, especially USP2), which regulate this membrane potential [19,121]. This suggestion is confirmed in the work [118], where the authors clearly show that the overexpression of UCP2 in XPA-deficient cells rescues the mitochondrial phenotype. Moreover, they checked an upstream regulatory axis: UCP2 is tightly regulated by transcription factor PGC-1α (peroxisome proliferator-activated receptorγ, coactivator 1), and PGC-1α activity is regulated by SIRT1 (a protein belonging to NAD^+^-dependent class III of histone deacetylases). They found that SIRT1 attenuation decreases mitophagy through the suppression of PGC-1α and UCP2. In mammalian cells, deacetylase SIRT1 is a crucial epigenetic regulator involved in cell metabolism, genomic stability maintenance, reprograming, aging, and tumorigenesis [122]. SIRT1-mediated deacetylation of PGC-1α increases its transcriptional activity [117]. Overall, SIRT1 may play a central role in mitophagy through the regulation of UCP2 via PGC-1α [118].

The SIRT1 activity requires cellular NAD^+^ and is effectively influenced by cellular metabolic and redox states [123]. A major cellular NAD^+^-consuming enzyme is PARP1 [124,125]. PARP1 is considered as one of damaged DNA sensors and key regulators of DNA repair and other cellular processes. In response to DNA damage in higher eukaryotes, nuclear proteins are modified by the poly(ADP-ribose) (PAR). PARylation is initiated by the transfer of an ADP-ribose unit from NAD^+^ to a target protein, and then PAR synthesis may proceed with successive additions of many ADP-ribose units giving rise to a large chain of a branched polymer. The PAR is thought to be the third type of nucleic acid, and some repair proteins can bind both free PAR and PAR attached to PARP1 noncovalently, as if it were DNA or RNA, via specific PAR-binding motifs [126].

Both PARP1 and SIRT1 use NAD^+^ for their activity and interact physically. Moreover, there is a strong connection between acetylation and PARylation. Under stressful conditions, PARP1 is acetylated, and this modification enhances its enzymatic activity. Nonetheless, SIRT1 may then deacetylase PARP1 and inhibit the PARP1 enzymatic activity. Under severe stress, PARP1 can become overactivated and may deplete cellular NAD, thereby repressing SIRT1 activity [127,128] and suppressing *SIRT1* transcription [129]. To prevent this situation, SIRT1 is also capable negatively regulating the expression of the *PARP1* gene [123].

**Figure 6:**
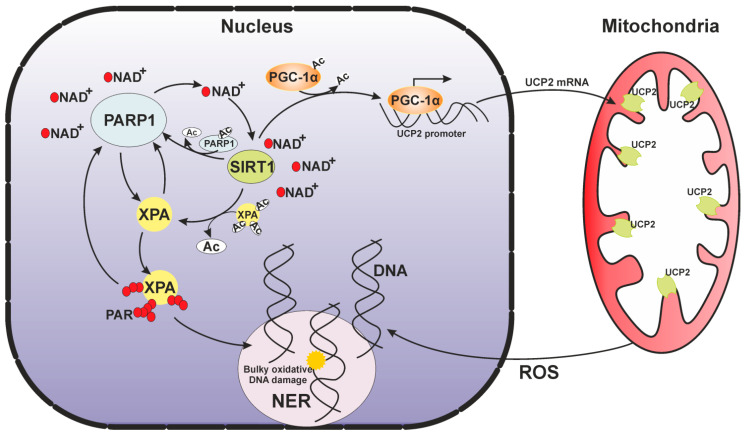
SIRT1–PARP1 reciprocal regulation within the nuclear–mitochondrial crosstalk occurs during normal cellular metabolism.

Thus, if we add XPA, which is a target of modifications by both PARP1 and SIRT1, to this sophisticated picture of reciprocal regulatory relations, we will see another link between DNA repair and a stress response (Figure 6). SIRT1 favors NER by deacetylating XPA and promoting subsequent XPA phosphorylation [118,130]. XPA has been found to be PARylated rapidly following UV irradiation, and this modification facilitates XPA recruitment to a site of DNA damage [131,132] but impairs its DNA-binding activity [132]. The XPA protein contains a conserved PAR-binding motif [132,133], that it is overlapped with DDB2 and TFIIH binding domains [134,135,136]. Thus, we can propose that PAR binding modulates the XPA interaction with these proteins and moreover influences (or even manages) XPA involvement in NER. In addition, XPA physically interacts with PARP1 and further stimulates the PARP1 activity. PARylation may strengthen the XPA–PARP1 interaction and promotes additional PARylation events. This mutual influence is combined with PARylation and is essential for the opening of chromatin and proficient NER [131,132]. When the XPA protein is mutated, this circle of reciprocal regulatory relations can be broken partially or completely (depending on the type of mutation) and therefore can contribute to the deterioration of cellular functions and eventually to the clinical features of XP.

To sum up, in XPA-deficient cells, a lack of NER activity lead to DNA damage accumulation. Under normal conditions, PARP1 activation is needed for cell homeostasis. Massive DNA damage triggers overactivation of PARP1 catalytic activity resulting in NAD^+^ intracellular depletion and the attenuation of SIRT1 activity. The inhibited SIRT1 cannot deacetylate PGC-1α, and its transcriptional activity stays switched off thus blocking the SIRT1–PGC-1α–UCP2 axis and causing a subsequent mitophagy defect. After that, the mitophagy defect leads to the accumulation of damaged mitochondria content that produce more ROS, which cause more DNA damage and more PARP1 activation and so on until cell death. As mentioned above, this pathological state could be reversed by PARP1 inhibition and restoration of the nuclear–mitochondrial crosstalk (particularly mitophagy) via overexpression of UCP2 and/or supplementation with a NAD^+^ precursor [118] in combination with antioxidant therapy [65].

## 7. Conclusions

Ideally, the design of therapeutic approaches requires that we understand relevant events step by step from a single molecule to the cell, tissue, and finally, the whole organism. Today, we know molecular mechanisms of DNA repair pathways in general and are beginning to understand the mechanisms of their regulation. This review indicates how much more there is to learn about the link between DNA repair deficiencies and real-world clinical features. We hope that biochemical and biophysical approaches developed in the future will help us to answer the question how to restore inadequate repair processes and to avoid DNA damage accumulation especially in neurons, as in XP and age-related diseases. Furthermore, it would be interesting to discover new therapeutic strategies against XP and treatments of age-related mitochondrial dysfunction. Of course, in the cancer treatment field, these research advances will be beneficial for the directed dysregulation of DNA repair in cancer cells to enhance the efficiency of DNA-damaging agents.

## Figures and Tables

**Table 1:** Comparison of clinical features of patients with XP, XP neurological disease, XP/CS, and CS [11,12].

Clinical Features	XP	XP Neurological Disease	XP/CS	CS
Molecular defects	XP-A, -B, -C, -D, -E, -F and XP-V	XP-A and XP-D followed by XP-B, XP-G, and XP-F	XP-B, XP-D, XP-G	CS-A, CS-B
Skin sun sensitivity	+	+	+	+
Increased freckling	+	+	+	-
Sunlight—induced skin cancer	+	+	+	-
Photophobia	+	+	+	+
Anterior eye cancer	+	+	+	-
Retinal degeneration	-	-	+	+
Sensorineural deafness	-	+	+	+
Developmental delay	-	+	+	+
Progressive neurological degeneration	-	+	+	+
Primary neuronal degeneration	-	+	-	-
Demyelination of brain	-	-	+	+
Cerebral atrophy	-	+	+	+
Cerebellar atrophy	-	+	+	+
Calcification (basal ganglia)	-	-	+	+

“+”—indicated clinical feature is associated with the disease; “-”—this clinical feature does not observed.

## Data Availability

The study did not report any data.
